# Oxidative stress and Parkinson’s disease

**DOI:** 10.3389/fnana.2015.00091

**Published:** 2015-07-08

**Authors:** Javier Blesa, Ines Trigo-Damas, Anna Quiroga-Varela, Vernice R. Jackson-Lewis

**Affiliations:** ^1^Centro Integral de Neurociencias A.C., HM Puerta del Sur, Hospitales de Madrid, Móstoles and Medical School, CEU San Pablo University, MadridSpain; ^2^Department of Medicine, Clinica Neurologica, Ospedale Santa Maria della Misericordia – Università di Perugia, PerugiaItaly; ^3^Department of Pathology and Cell Biology, Columbia University, New York, NYUSA

**Keywords:** mitochondrial dysfunction, dopamine, neuroinflammation, Parkinson disease, oxidative stress

## Abstract

Parkinson disease (PD) is a chronic, progressive neurological disease that is associated with a loss of dopaminergic neurons in the substantia nigra pars compacta of the brain. The molecular mechanisms underlying the loss of these neurons still remain elusive. Oxidative stress is thought to play an important role in dopaminergic neurotoxicity. Complex I deficiencies of the respiratory chain account for the majority of unfavorable neuronal degeneration in PD. Environmental factors, such as neurotoxins, pesticides, insecticides, dopamine (DA) itself, and genetic mutations in PD-associated proteins contribute to mitochondrial dysfunction which precedes reactive oxygen species formation. In this mini review, we give an update of the classical pathways involving these mechanisms of neurodegeneration, the biochemical and molecular events that mediate or regulate DA neuronal vulnerability, and the role of PD-related gene products in modulating cellular responses to oxidative stress in the course of the neurodegenerative process.

## Introduction

Parkinson’s disease (PD) is associated with the selective loss of dopamine (DA) neurons in the substantia nigra pars compacta (SNpc) and DA levels in the corpus striatum of the nigrostriatal DA pathway in the brain. This loss of DA causes a deregulation in the basal ganglia circuitries that leads to the appearance of motor symptoms such as bradykinesia, resting tremor, rigidity, and postural instability as well as non-motor symptoms such as sleep disturbances, depression, and cognitive deficits (Rodriguez-Oroz et al., 2009). The exact etiology of PD still remains elusive and the precise mechanisms that cause this disease remain to be identified (Obeso et al., 2010). At the cellular level, PD is related to excess production of reactive oxygen species (ROS), to alterations in catecholamine metabolism, to modifications in mitochondrial electron transporter chain (METC) function or to enhancement of iron deposition in the SNpc. The failure of normal cellular processes that occur in relation to the aging process are also believed to contribute to the increased vulnerability of DA neurons (Schapira and Jenner, 2011; Rodriguez et al., 2014).

While the familial forms of PD, that have been described, involve mutations in a number of genes (Kieburtz and Wunderle, 2013; Trinh and Farrer, 2013), mitochondrial dysfunction, neuroinflammation and environmental factors are increasingly appreciated as key determinants of dopaminergic neuronal susceptibility in PD, and are a feature of both familial and sporadic forms of the disease (Ryan et al., 2015). In both cases, oxidative stress is thought to be the common underlying mechanism that leads to cellular dysfunction and, eventual cell death. ROS are continuously produced *in vivo* by all body tissues. However, oxidative stress occurs when there is an imbalance between ROS production and cellular antioxidant activity. Oxidants and superoxide radicals are produced as products of oxidative phosphorylation, making mitochondria the main site of ROS generation within the cell. ROS can affect mitochondrial DNA which can cause modulations in the synthesis of METC components like adenosine triphosphate (ATP) production as well as the leakage of ROS into the cell’s cytoplasm (Brieger et al., 2012).

**FIGURE 1:**
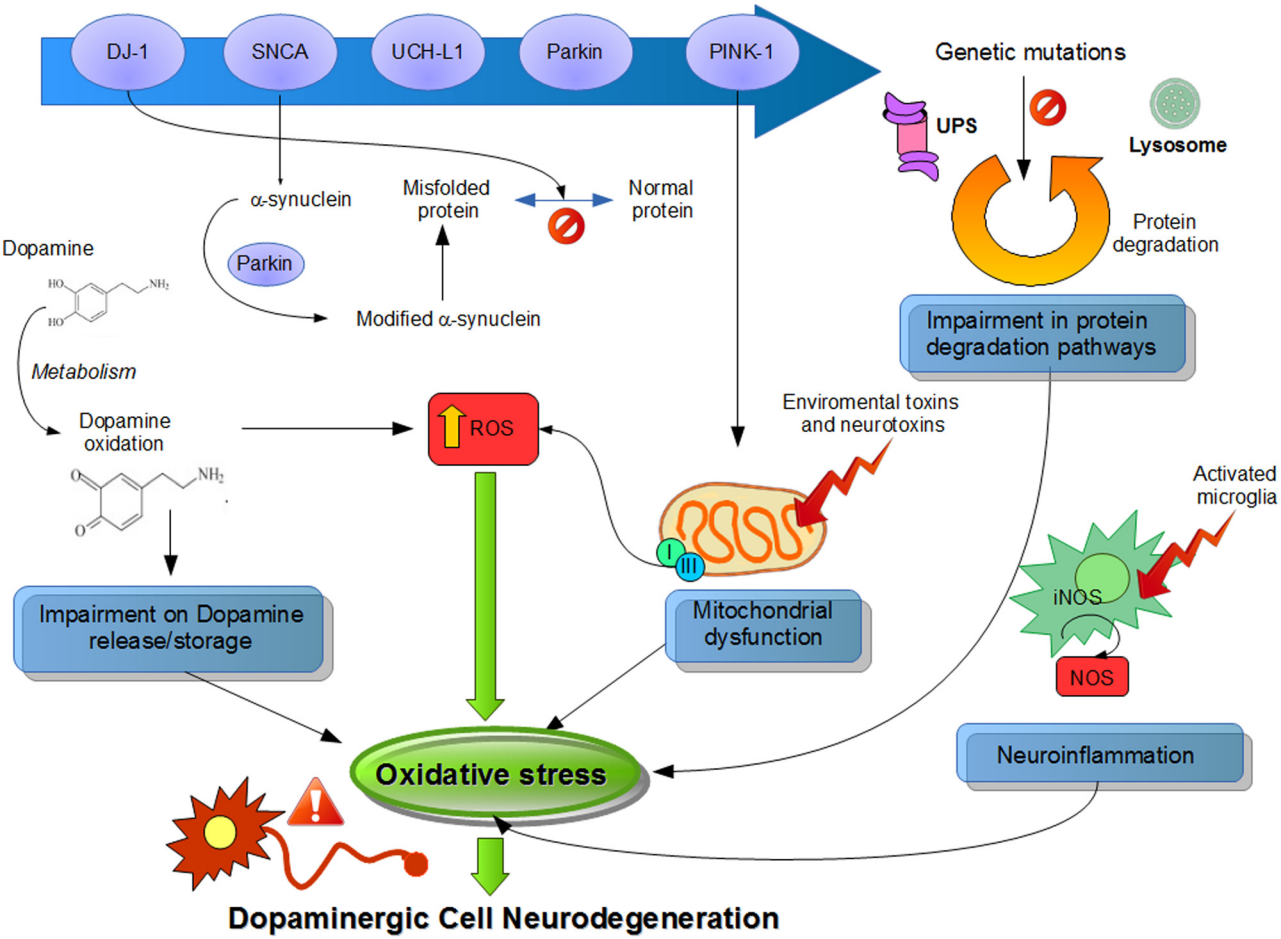
**Suggested physiological processes related to pathogenesis of Parkinson’s disease (PD).** Different pathways and their dysfunctions resulting from genetic modifications in PD-related genes and lead to an increased oxidative stress. Mutations or altered expression of these proteins result in mitochondrial impairment, oxidative stress, and protein misfolding. Also, dopamine metabolism may be oxidized to reactive dopamine quinones contributing to increased levels of reactive oxygen species. α-Synuclein becomes modified and accelerate its aggregation. Increased oxidative stress provokes impaired function of the UPS that degrades misfolded or damaged proteins and hereby further affecting cell survival. Environmental toxins impair mitochondrial function, increase the generation of free radicals, and lead to aggregation of proteins, including α-synuclein. Mitochondrial dysfunction by complex I inhibition affects by adding an increase in oxidative stress and a decline in ATP production, leading to damage of intracellular components and to cell death. Also, neuroinflammatory mechanisms might contribute to the cascade of consequences leading to cell death. In summary, all these several cellular mechanisms attributed to oxidative stress are implicated in the selective degeneration of dopaminergic neurons.

Although the precise mechanism corresponding to ROS generation related to PD is still unknown, in this review, we summarize the major sources of oxidative stress generated by the DA neurons, like DA metabolism, mitochondrial dysfunction, and neuroinflammation (**Figure 1**).

## Dopamine Metabolism

Selective degeneration of the DA neurons of the SNpc suggests that DA itself may be a source of oxidative stress (Segura-Aguilar et al., 2014). DA is synthesized from tyrosine by tyrosine hydroxylase (TH) and aromatic amino acid decarboxylase. Following this, DA is stored in synaptic vesicles after uptake by the vesicular monoamine transporter 2 (VMAT2). However, when there is an excess amount of cytosolic DA outside of the synaptic vesicle in damaged neurons, i.e., after L-DOPA treatment, DA is easily metabolized via monoamine oxidase (MAO) or by auto-oxidation to cytotoxic ROS (Zucca et al., 2014). For example, mishandling of DA in mice with reduced VMAT2 expression was sufficient to cause DA-mediated toxicity and progressive loss of DA neurons (Caudle et al., 2007).

This oxidative process alters mitochondrial respiration and induces a change in the permeability transition pores in brain mitochondria (Berman and Hastings, 1999). Also, the auto-oxidation of DA produces electron-deficient DA quinones or DA semiquinones (Sulzer and Zecca, 2000). Some studies have demonstrated a regulatory role for quinone formation in DA neurons in the L-DOPA-treated PD model induced by neurotoxins and in methamphetamine neurotoxicity (Asanuma et al., 2003; Miyazaki et al., 2006; Ares-Santos et al., 2014). DA quinones can modify a number of PD-related proteins, such as α-synuclein (α-syn), parkin, DJ-1, Superoxide dismutase-2 (SOD2), and UCH-L1 (Belluzzi et al., 2012; Girotto et al., 2012; da Silva et al., 2013; Hauser et al., 2013; Toyama et al., 2014; Zhou et al., 2014) and have been shown to cause inactivation of the DA transporter (DAT) and the TH enzyme (Kuhn et al., 1999; Whitehead et al., 2001), as well as mitochondrial dysfunction (Lee et al., 2003), alterations of brain mitochondria (Gluck and Zeevalk, 2004) and dysfunction in Complex I activity (Jana et al., 2007, 2011; Van Laar et al., 2009). Additionally, DA quinones can be oxidized to aminochrome, whose redox-cycling leads to the generation of the superoxide radical and the depletion of cellular nicotinamide adenine dinucleotide phosphate-oxidase (NADPH), which ultimately forms the neuromelanin (Sulzer et al., 2000) known to be accumulated in the SNpc of the human brain (Ohtsuka et al., 2013, 2014; Plum et al., 2013).

Significant increases in cysteinyl adducts of L-DOPA, DA, and DOPAC have been found in substantia nigra of PD patients, suggesting the cytotoxic nature of DA oxidation (Spencer et al., 1998). Also, DA terminals actively degenerated proportionally to increased levels of DA oxidation following a single injection of DA into the striatum (Rabinovic et al., 2000). Recently, it has been shown that increased uptake of DA through the DAT in mice results in oxidative damage, neuronal loss and motor deficits (Masoud et al., 2015).

## Mitochondrial Dysfunction

Mitochondrial dysfunction is closely related to increased ROS formation in PD (Schapira, 2008). Oxidative phosphorylation is the main mechanism providing energy to power neural activity in which the mitochondria use their structure, enzymes, and energy released by the oxidation of nutrients to form ATP (Hall et al., 2012). Consequently, this metabolic pathway is the main source of superoxide and hydrogen peroxide, which, at the same time, lead to propagation of free radicals contributing to the disease.

Complex I deficiencies of the respiratory chain account for the majority of unfavorable neural apoptosis generation and is considered one of the primary sources of ROS in PD. Complex I inhibition results in an enhanced production of ROS, which, in turn, will inhibit complex I. Reduction in complex I activity in the SNpc of patients with sporadic PD has been well described (Schapira et al., 1990; Hattori et al., 1991; Hattingen et al., 2009). Additionally, mitochondrial complex I deficiency in different brain regions (Mizuno et al., 1989; Parker et al., 2008), fibroblasts (Mytilineou et al., 1994), blood platelets (Krige et al., 1992; Blandini et al., 1998), skeletal muscle (Blin et al., 1994), and lymphocytes (Yoshino et al., 1992; Haas et al., 1995) of PD patients has been shown before as well.

As such, complex I inhibitors like 1-methyl-4-phenyl-1,2,3,6-tetrahydropyridine (MPTP) or rotenone show preferential cytotoxicity to the DA neurons (Blesa and Przedborski, 2014). The mechanism by which MPTP crosses the blood–brain barrier and is oxidized to 1-methyl-4-phenylpyridinium (MPP+) is well known (Blesa and Przedborski, 2014). The MPP+ accumulates in the mitochondria where it inhibits complex I in the METC, therefore disrupting the flow of electrons along the METC, which results in decreased ATP production and increased generation of ROS (Mizuno et al., 1987). Like MPTP, rotenone is another mitochondrial complex I inhibitor. Interestingly, rotenone toxicity is involved in oxidative damage to proteins and Lewy body-like inclusions (Betarbet et al., 2000; Sherer et al., 2003a,b; Greenamyre et al., 2010). The events downstream to complex I inhibition that lead to neuronal cell death by these toxins are still unknown (Schapira, 2010).

Other evidence for mitochondrial dysfunction related to oxidative stress and DA cell damage comes from findings that mutations in genes of proteins like α-syn, parkin, DJ-1, or PINK are linked to familial forms of PD. The convergence of all of these proteins on mitochondrial dynamics uncovers a common function in the mitochondrial stress response that might provide a potential physiological basis for the pathology of PD (Norris et al., 2015; van der Merwe et al., 2015). Overall, these observations show that mutations in these genes affect mitochondrial function and integrity and, are associated with increases in oxidative stress (Zuo and Motherwell, 2013). ROS influence proteasomal, lysosomal, and mitochondrial function, which, in turn, regulate the cellular response to oxidative damage (Cook et al., 2012). The correct elimination of damaged proteins by effective proteolysis and the synthesis of new and protective proteins are vital in the preservation of brain homeostasis during periods of increased levels of ROS. Consequently, this can lead to protein misfolding (i.e., α-syn), preventing the ability of some of these proteins to be unfolded and degraded by the systems that regulate protein clearance, like the ubiquitin proteasome system or autophagy. Indeed, protein misfolding, together with the dysfunction of these protein degradation systems, may play a key role in the appearance of deleterious events implicated in the neurodegenerative process of PD (Schapira et al., 2014).

Parkin and PINK1 are localized in the mitochondria and their functions are tightly connected to the normal functioning of the mitochondria (Scarffe et al., 2014). PINK1 accumulates on the outer membrane of damaged mitochondria and recruits Parkin to the dysfunctional mitochondrion (Pickrell and Youle, 2015). In humans with parkin mutations, mitochondrial complex I activity is impaired (Müftüoglu et al., 2004). Overexpression of parkin in mice reduced DA neuronal cell loss induced by MPTP through the protection of mitochondria and the reduction of α-syn (Bian et al., 2012). On the other hand, parkin KO mice showed decreased amounts of several proteins that are involved in mitochondrial function and oxidative stress as well as increases in protein oxidation and lipid peroxidation (Palacino et al., 2004). Also, *Drosophila*, lacking, or deficient in parkin, exhibit mitochondrial deficits and high vulnerability to oxidative stress (Saini et al., 2010). PINK1 mutations in humans lead to mitochondrial defects and respiratory chain abnormalities (Hoepken et al., 2007; Piccoli et al., 2008). PINK1 KO in human and mouse DA neurons causes decreases in membrane potential and increases in ROS generation (Wood-Kaczmar et al., 2008). The decrease in mitochondrial membrane potential is not due to a proton leak, but to respiratory chain defects like complex I and complex III deficiency (Amo et al., 2011, 2014). Therefore, PINK1 is required for maintaining normal mitochondrial morphology of SNpc DA neurons in culture and exerts its neuroprotective effect by inhibiting ROS formation (Wang et al., 2011). In animal models, studies show that the lack of PINK1 resulted in abnormal mitochondrial morphology, loss of SNpc DA neurons, reduction in complex I activity, and enhanced vulnerability to oxidative stress (Clark et al., 2006; Kitada et al., 2007; Gautier et al., 2008). These defects can be ameliorated and rescued by the enhanced expression of parkin (Yang et al., 2006; Exner et al., 2007). This last scenario seems to involve PINK1 and Parkin in a common pathway that regulates mitochondrial physiology and cell survival in which PINK1 seems to be functioning upstream of Parkin, at least as observed in *Drosophila* disease models (Clark et al., 2006).

α-syn is a soluble protein that is highly enriched in the presynaptic terminals of neurons. Accumulation of α-syn as intracellular filamentous aggregates is a pathological feature of both sporadic and familial PD (Goedert et al., 2013). Accumulation of wild-type α-syn in DA neurons reduced mitochondrial complex I activity, elevated ROS production leading to cell death (Martin et al., 2006). It has been shown that α-syn inclusions elevate dendritic mitochondrial oxidative stress in DA neurons (Dryanovski et al., 2013). This mitochondrial dysfunction occurs many months before the occurrence of striatal DA loss (Subramaniam et al., 2014). The nuclear translocation of α-syn increases susceptibility of MES23.5 cells to oxidative stress (Zhou et al., 2013). Exposure to rotenone or other stimuli that promote ROS formation and mitochondrial alterations correlate well with mutant α-syn phosphorylation at Ser129 (Perfeito et al., 2014). Oxidative stress promotes uptake, accumulation, and oligomerization of extracellular α-syn in oligodendrocytes (Pukass and Richter-Landsberg, 2014) and induces posttranslational modifications of α-syn which can increase DA toxicity (Xiang et al., 2013). It has been suggested that the NADPH oxidases, which are responsible for ROS generation, could be major players in synucleinopathies (Cristóvão et al., 2012).

DJ-1 is another gene reported to cause a familial early onset PD (Puschmann, 2013). DJ-1 binds to subunits of mitochondrial complex I and regulates its activity (Hayashi et al., 2009). Although a portion of DJ-1 is present in mitochondria matrix and inter-membrane space (Zhang et al., 2005), the degree of translocation of DJ-1 into mitochondria is stimulated by oxidative stress (Canet-Avilés et al., 2004). Mitochondrial-targeted sequence-conjugated DJ-1 has been shown to be more protective against oxidative stress-induced cell death (Junn et al., 2009). DJ-1 KO mice displayed nigrostriatal DA neuron loss (Goldberg et al., 2005). Also, these DJ-1 KO mice showed altered mitochondrial respiration and morphology, reduced membrane potential, and accumulation of defective mitochondria (Irrcher et al., 2010; Krebiehl et al., 2010; Giaime et al., 2012). These defects can be reversed by DJ-1 overexpression, which points to the specific role of DJ-1 in mitochondrial function (Heo et al., 2012). Recently, following oxidative stress, DJ-1 was shown to be involved in the oxidative stress response that leads to the upregulation of the proteasome, thus inhibiting its activity and rescuing partially unfolded proteins from degradation (Moscovitz et al., 2015).

## Neuroinflammation

Neuronal loss in PD is associated with chronic neuroinflammation, which is controlled primarily by microglia, the major resident immune cells in the brain (Barcia et al., 2003) and, to a lesser extent, by astrocytes and oligodendrocytes (Perry, 2012). Microglial activation has been found with a greater density in the SNpc (Lawson et al., 1990) and in the olfactory bulb of both sporadic and familial PD patients (McGeer et al., 1988; Doorn et al., 2014a,b). Additionally, activated microglia have been found in the SNpc and in the striatum of PD animal models (Pisanu et al., 2014; Stott and Barker, 2014) and have been associated with different PD-associated gene/proteins like *α-syn* or *LRRK2* (Daher et al., 2014; Sacino et al., 2014). In response to certain environmental toxins and endogenous proteins, microglia can shift to an over-activated state and release ROS which can cause neurotoxicity (Block et al., 2007). Accumulating evidence indicates that activation of different enzymes like NADPH oxidase (NOX2) in microglia is neurotoxic not only through the production of extracellular ROS that damage neighboring neurons but also through the initiation of redox signaling in microglia that amplifies the pro-inflammatory response (Surace and Block, 2012).

Neuromelanin confers the dark pigmentation that is produced from DA oxidation and is so characteristic of the SNpc appearance. High levels of catecholamine metabolism in the midbrain are associated with increased levels of neuromelanin in the same region and, it is neuromelanin that is thought to be one of the molecules responsible for inducing chronic neuroinflammation in PD. Neuromelanin released from dying DA neurons in the SNpc activate microglia, increasing the sensitivity of DA neurons to oxidative stress-mediated cell death (Halliday et al., 2005; Li et al., 2005; Beach et al., 2007; Zhang et al., 2009). The ability of neuromelanin to interact with transition metals, especially iron, and to mediate intracellular oxidative mechanisms have received particular attention. Increased levels of iron result in increased ROS and increased oxidative stress and has been shown to be involved in aging and PD. Iron homeostasis is modulated by angiotensin in DA neurons and microglia, and glial cells play an essential role in the efficient regulation of this balance (Garrido-Gil et al., 2013).

Dopamine neurons containing neuromelanin are especially more susceptible, indicating a possible role for neuromelanin in MPTP-toxicity (Herrero et al., 1993). MPTP induces a glial response, increased levels of inflammatory cytokines and microglial activation in mice (Członkowska et al., 1996; Jackson-Lewis and Smeyne, 2005) and monkeys (Barcia et al., 2004, 2009). Angiotensin is one of the most important inflammation and oxidative stress inducers, and produces ROS by activation of the NADPH-oxidase complex. It has been suggested that the inflammatory response in the MPTP model could be mediated by brain angiotensin and microglial NADPH-derived ROS (Joglar et al., 2009). Moreover, oral treatment with NADPH oxidase antagonists mitigates the clinical and pathological features of parkinsonism in the MPTP marmoset model (Philippens et al., 2013). Also, microglia play an important role in mediating rotenone-induced neuronal degeneration through NADPH (Gao et al., 2003, 2011; Pal et al., 2014). Rotenone increased microglial activation in both the SNpc and striatum in rats (Sherer et al., 2003a), activated microglia via the NF-κB signaling pathway (Gao et al., 2013) and induced neuronal death by the microglial phagocytosis of neurons (Emmrich et al., 2013).

Parkinson’s disease-associated proteins like α-syn, parkin, LRRK2, and DJ-1 have also been reported to activate microglia (Wilhelmus et al., 2012). Extracellular α-syn released from neuronal cells is an endogenous agonist for Toll-like receptor 2 (TLR2), which activates the microglial inflammatory responses (Kim et al., 2013a). An increased number of activated microglia and increased levels of TNF-α mRNA and protein were detected in the striatum and in the SNpc of mice over-expressing WT human α-syn (Watson et al., 2012). Moreover, in α-syn KO mice, microglia secreted higher levels of proinflammatory cytokines, TNF alpha and IL-6 (interleukin-6) compared to WT mice (Austin et al., 2006). Intracerebral injection of recombinant amyloidogenic or soluble α-syn induces extensive α-syn intracellular inclusion pathology that is associated with a robust gliosis (Sacino et al., 2014). LRRK2 increases proinflammatory cytokine release from activated primary microglial cells which results in neurotoxicity (Gillardon et al., 2012). In contrast, LRRK2 inhibition attenuates microglial inflammatory responses (Moehle et al., 2012). Additionally, lipopolysaccharide induces LRRK2 up-regulation and microglial activation in mouse brains (Li et al., 2014) but they down regulated Parkin expression via NF-κB (Tran et al., 2011). Abnormal glial function is critical in parkin mutations, increasing vulnerability to inflammation-related nigral degeneration in PD (Frank-Cannon et al., 2008) and its role increases with aging (Solano et al., 2008). DJ-1 expression is up-regulated in reactive astrocytes in PD patients (Bandopadhyay et al., 2004). DJ-1 negatively regulates inflammatory responses of astrocytes and microglia by facilitating the interaction between STAT1 and its phosphatase SHP-1 (Kim et al., 2013b). Astrocyte cultures from DJ-1 KO mice treated with lipopolysaccharide have increased NO production and an up-regulation of different pro-inflammatory mediators like COX-2 and IL-6 (Waak et al., 2009).

## Conclusion

The elements that potentially cause oxidative stress in PD are still unknown. DA metabolism, mitochondrial dysfunction and neuroinflammation all play critical roles in the etiology of this disease. Exposure to environmental factors or mutations in PD-associated genes of patients with either sporadic or familial PD may cause mitochondrial dysfunction that ultimately results in PD. All of these share common linkages and influence each other greatly. Limiting the early inflammatory response will reduce further both elevated oxidative stress and microglial activation that are key to slowing the death of the neurons in the SNpc. Development of potential drugs able to delay the neurodegenerative process is crucial to ameliorating the deleterious effects of oxidative stress in neurodegenerative diseases. Neuroprotective therapies will need to target multiple pathological pathways such as mitochondrial dysfunction and neuroinflammation in the next few years.

## Conflict of Interest Statement

The authors declare that the research was conducted in the absence of any commercial or financial relationships that could be construed as a potential conflict of interest.
